# The identification and validation of EphA7 hypermethylation, a novel biomarker, in cervical cancer

**DOI:** 10.1186/s12885-022-09653-7

**Published:** 2022-06-09

**Authors:** Wenfan Zhang, Huiling Cao, Jinhao Yang, Jing Zhao, Zheng Liang, Xiaodong Kang, Rong Wang

**Affiliations:** 1 Department of Laboratory Medicine, Tianjin Medical University, Tianjin, China; 2 Department of Gynecology and Obstetrics, Tianjin Medical University General Hospital, Tianjin, China; 3 Department of Otorhinolaryngology, Tianjin Medical University General Hospital, Tianjin, China; 4 Department of Medical Image, Tianjin Medical University, Tianjin, China

**Keywords:** EphA7, DNA methylation, CRISPR-dCas9, Cervical cancer

## Abstract

**Background:**

Aberrant methylation of EphA7 has been reported in the process of carcinogenesis but not in cervical cancer. Therefore, an integration study was performed to explore the association between EphA7 hypermethylation and cervical cancer and validate the potential value of EphA7 hypermethylation in the diagnosis of cervical cancer.

**Methods:**

We performed an integration study to identify and validate the association between EphA7 methylation and cervical cancer. First, data on EphA7 methylation and expression in cervical cancer were extracted and analyzed via bioinformatics tools. Subsequently, CRISPR-based methylation perturbation tools (dCas9-Tet1/DNMT3a) were constructed to further demonstrate the association between DNA methylation and EphA7 expression. Ultimately, the clinical value of EphA7 methylation in cervical cancer was validated in cervical tissues and Thinprep cytologic test (TCT) samples by methylation-specific PCR (MSP) and quantitative methylation-specific PCR (QMSP), respectively.

**Results:**

Pooled analysis showed that EphA7 promoter methylation levels were significantly increased in cervical cancer compared to normal tissues (*P* < 0.001) and negatively correlated with EphA7 expression. These prediction results were subsequently confirmed in cell lines; moreover, CRISPR-based methylation perturbation tools (dCas9-Tet1/DNMT3a) demonstrated that DNA methylation participates in the regulation of EphA7 expression directly. Consistent with these findings, the methylation level and the positive rate of EphA7 gradually increased with severity from normal to cancer stages in TCT samples (*P* < 0.01).

**Conclusions:**

EphA7 hypermethylation is present in cervical cancer and is a potential biomarker for the diagnosis of cervical cancer.

**Supplementary Information:**

The online version contains supplementary material available at 10.1186/s12885-022-09653-7.

## Background

Cervical cancer (CC) is the fourth most commonly diagnosed cancer and the fourth leading cause of cancer death in women. Approximately 604,000 new CC cases and 342,000 deaths were recorded worldwide in 2020 [1]. Recently, the World Health Organization (WHO) called for global action toward the elimination of CC by 2030 [2]. This is a challenge for China, which had an estimated 109,741 new cases and 59,060 deaths in 2020 according to International Agency for Research on Cancer (IARC) (https://www.iarc.fr/faq/latest-global-cancer-data-2020-qa/). Cervical carcinogenesis is a complex process with multiple factors and stages [3]. In addition to high-risk human papillomavirus (hr-HPV) infection, epigenetic abnormalities, especially alterations in DNA methylation, are involved in the development of CC [4, 5].

Eph receptors represent the largest family of receptor tyrosine kinases. In addition to its physiological roles, recent studies revealed that some Eph genes are associated with human malignancies [6]. For example, EphA1 and EphA4 are upregulated in gastric cancer [7]; EphA2 overexpression in mammary epithelial cells induces tumorigenesis [8] and EphA8 stimulates the proliferation, invasion, and migration of gastric cancer cells [9].

As a member of the Eph receptor group, EphA7 is associated with carcinogenesis [10] but plays a contradictory role in different cancers [11–14]. EphA7 is overexpressed in hepatocellular carcinoma [15], glioblastoma multiform [11], gallbladder adenocarcinoma [16] and lung carcinoma [17] and contributes to malignant transformation, aggressive progression, and poor prognosis. However, EphA7 may also act as a tumor suppressor since EphA7 downregulation is induced by promoter hypermethylation in prostate cancer patients [18]. Similar results have also been observed in colorectal cancer [20], and oral squamous cell carcinoma [21] etc. Wang J [19, 20]confirmed hypermethylation of the promoter of EphA7 in gastric carcinoma since EphA7 expression was restored after demethylation treatment with 5-aza-2-deoxycytidine (5-aza-dc). However, the link between EphA7 and CC remains unclear. Therefore, this study aims to reveal the association of EphA7 methylation with CC and to validate the potential value of EphA7 methylation in the clinical diagnosis of CC.

## Methods

### Pooled analysis of databases and web tools

Gene expression and DNA methylation data of EphA7 in cervical tissues were extracted from The Cancer Genome Atlas (TCGA, https://cancergenome.nih.gov/) and Genotype-Tissue Expression (GTEx, https://commonfund.nih.gov/GTEx) databases. EphA7 expression data were collected from GEPIA (http://gepia2.cancer-pku.cn/) [22] based on TCGA and GTEx. The web tools UALCAN (http://ualcan.path.uab.edu/) [23] and Wanderer (http://maplab.imppc.org/wanderer/) [24] were utilized to compare the level of methylation between adjacent and tumor tissues. The relationships between EphA7 expression and methylation were evaluated by MEXPRESS (https://mexpress.be/) [25]. JASPAR (http://jaspar.genereg.net/) [26] was applied to predict the transcription factors that bind to the promoter of EphA7. The relationship between EphA7 methylation and the survival time was analyzed by LinkedOmics (http://www.linkedomics.org/login.php) [27] and the expression with the survival was performed through Human Protein Atlas (https://www.proteinatlas.org/) [28].

### Cell lines and clinical sample collection

#### Cell culture

Human CC cell lines, including CaSki and SiHa, and a human embryonic kidney cell line were purchased from Zhong Qiao Xin Zhou (Shanghai, China).The cell lines were tested for mycoplasma by PCR and were authenticated using STR profiling. The above 3 cell lines were cultured in RPMI 1640, MEM and DMEM (HyClone, USA) supplemented with 10% fetal bovine serum (BI, USA) and 1% penicillin–streptomycin (Solarbio, China) at 37 °C in 5% CO_2_ and saturated humidity.

#### Clinical specimens

Cervical frozen tissues and Thinprep cytologic test (TCT) specimens were obtained from the Department of Gynecology and Obstetrics, Tianjin Medical University General Hospital, from January 2016 to June 2019. This study was approved by the Medical Ethics Committee of Tianjin Medical University, and followed the Declatation of Helsinki on biology for human trials. All patients gave informed consent.None of the patients received radiotherapy or chemotherapy prior to surgery. According to the results of the pathological diagnosis, patients in the study group were divided into normal, cervical intraepithelial neoplasia II (CINII), CINIII and cancer groups.

A total of 57 frozen tissue specimens were collected for testing. The frozen tissue samples consisted of 25 normal, 24 CIN II/III and 8 cancer samples and were stored at -80 °C. In addition, 114 TCT specimens were collected, including 28 normal cervical samples, 24 CINII grade specimens, 45 CINIII grade specimens and 17 CC specimens. The specimens were obtained using a disposable cervical specimen collection brush and stored in TCT preservation solution (BD Surepath, USA) at 4 °C.

### Selection of single guide RNA(sgRNA) sequences and creation of U6-sgRNA PCR cassettes

The sgRNAs were designed to target the EphA7 CpG island of interest using the public tool CRISPR-ERA (http://crispr-era.stanford.edu/) as previously described [29]. U6-sgRNA PCR cassettes were created from an sgRNA vector (#84477) by using primers (Table. 1) that amplify a U6 promoter fused to the sgRNA reverse complement and sgRNA forward complement fused to the remaining sgRNA scaffold cassette, followed by another round of overlap-extension PCR to amplify the full U6-sgRNA PCR cassette. Fuw-dCas9-Tet1CD (#84475), Fuw-dCas9-Tet1CD_IM (#84479), Fuw-dCas9-DNMT3a (#84476) and Fuw-dCas9-DNMT3a_IM (#84478) plasmids were purchased from Addgene [30]. Three active sgRNAs (act-sgRNAs) for demethylation and 4 repressive sgRNAs (rep-sgRNAs) for increasing methylation were designed and are listed in Table. 1; Please see Figure. s1 for the gene map and sgRNA sites.

**Table 1:** sgRNA detailed information for EphA7 via CRISPR-ERA

		**Sequence**	**Distance to TSS(bp)**	**Strand**
**Acitivation**	act-sgRNA1	GCGCGAGCTCAGAACCTGGA	-209	+
	act-sgRNA2	GGTCCGAGGCAGGAGCCAAT	-150	-
	act-sgRNA3	GGAATCGCCTCCTGGCAGGC	-78	+
**Repression**	rep-sgRNA1	GCAAGCGGCCGGTCTGCAGT	+33	+
	rep-sgRNA2	GTTTCAGTTATCTTGAGTCG	+216	-
	rep-sgRNA3	GCCGATCGGGGACCGAGAAG	+130	+
	rep-sgRNA4	GCAAGTCTCCGACTGCAGAC	+44	-

### Transient transfection

dCas9-Tet1 or dCas9-DNMT3a plasmids with U6-sgRNA cassettes were transfected into cells, including CaSki, SiHa and HEK293T, using OMNIfect Transfection Reagent (Transomic Technologies, USA) according to the manufacturer’s instructions. The ratio of dCas9-Tet1/DNMT3a to an individual sgRNA was 1:1. For the experiments in which sgRNAs were transfected together, the amount of each sgRNA was equal to aliquoted parts of the total amount of sgRNA. The mutated plasmids of dCas9-Tet1CD-IM (Tet1^m^) or dCas9-DNMT3a-IM (DNMT3a^m^) were considered as negative controls.

### RNA extraction and quantitative real-time PCR (qRT-PCR)

The harvested cells were dissolved in TRIzol® reagent (Invitrogen, USA), and total mRNA was then extracted following the manufacturer's protocol. qRT-PCR was performed using SYBR Green PCR Mix (Tiangen, China) and a Stratagene Mx3005P sequence detection system (StrataGene, Agilent, USA). The amplification parameters were as follows: 95 °C for 15 min, followed by 40 cycles at 95 °C for 10 s, 60 °C for 20 s, and 72 °C for 20 s. The 2^−∆∆Ct^ method was performed with the GAPDH gene as an internal control, and the relative quantification procedure was selected. The primer sequences are listed in Table s1.

### DNA extraction and bisulfite treatment

Genomic DNA from frozen tissues was extracted using a TIANamp Genomic DNA Kit (Tiangen Biotech, Beijing, China) according to the manufacturer's instructions. DNA from cervical liquid-based cell specimens was isolated by phenol/chloroform extraction. One microgram of genomic DNA per sample was modified using the EZ DNA methylation kit (Zymo Research Corp, Irvine, US) according to the manufacturer's instructions. Leukocyte DNA from healthy women was used as a negative control for methylation, while in vitro methylated leukocyte DNA produced using M. SssI methyltransferase (New England Biolabs, Ipswitch, USA) was used as a positive control.

### MSP (Methylation-specific PCR)

Primers were designed via Methy Primer Express v1.0 (Applied Biosystems, USA) and synthesized in Sangon Biotech (Shanghai, China).The primers are shown in Table s1. In total, 1.5 µl bisulfite-treated DNA were amplified in a 30 µl reaction mixture consisting of 1 × PCR Buffer with 0.5U AmpliTaq Gold DNA polymerase (Applied Biosystems, USA), 0.2 mM dNTP mix, and 0.3 µM of each primer. The PCR conditions were as follows: 95 °C for 10 min, then 40 cycles of 95 °C for1 min, 55 °C for 1 min, 72 °C for 1 min and finally an elongation step of 7 min at 72 °C. The PCR products were separated on a 2% agarose gel, prestained with Gelred (Shanghai Life iLab Bio, China) and visualized by UV transillumination. Leukocyte (leu) DNA from healthy women was used as a negative control, and in vitro methylated (iv) leukocyte DNA was used as a positive control.

### QMSP (Quantitative methylation-specific PCR)

Quantitative methylation-specific PCR was performed with a double-quenched (FAM/IBHQ)-labelled hybridization probe. The methylated primers for QMSP were the same as those used for MSP. The probes were designed using Clone Manager 9.0 software and synthesized by Sangon Biotech (Table s1). Twenty-five nanograms bisulfite-converted DNA was utilized for PCR with 0.3 µM primers, 0.2 µM probed and 1 × QuantiTect Probe PCR Master Mix (Qiagen, Germany) in 10 µl reaction for 50 cycles in a 7900HT Fast RealTime PCR System (Applied Biosystems). The ACTB gene was used as a methylation-independent internal reference gene. The criteria for the interpretation of positive methylation results were as follows: *Ct *value < 50 (at least 2 of 3 multiple wells) with sufficient methylated DNA (200 pg DNA). The relative level of EphA7 methylation was analyzed as previously described [31].

### Pyrosequencing

Pyromark PCR and sequencing primers were designed and generated according to the instructions of PyroMark Assay Design 2.0 (Table. s1). PCR amplification was carried out and optimized using a PyroMark® PCR Kit (Qiagen, Hilden, Germany) in a total reaction volume of 25 μl. The PCR volume was 25 µl with 0.2 µM primer mix, 1 × PyroMark master mix, 1 × CoralLoad Concentrate and 1 µl bisulfite-modified DNA. PCR testing was carried out at 95 °C for 10 min, followed by 45 cycles (94 °C for 30 s, 55 °C for 30 s, and 72 °C for 30 s, with a final extension at 72 °C for 10 min). The obtained PCR products were then subjected to pyrosequencing (PyroMark Q24 system; QIAGEN) on the PyroMark Q24 platform.

### Statistical analysis

Statistical analysis was performed using IBM SPSS Statistics 22.0 (IBM Corporation, New York, USA) and Graphpad Prism8.0 (GraphPad Software, USA).Students’ *t* test was used to compare differences in median methylation or expression levels between two groups conforming to normal distribution,whereas a Mann–Whitney U test was used for data not conforming to normal distribution. The correlation between the expression and methylation level was investigated via Pearson correlation coefficients. All transfections were done in triplicate, and for each biological replicate, at least three technical replicates of the qRT-PCR assay were performed. The Kaplan–Meier method was used for survival analysis, and a log-rank test was applied for comparations between groups.A *P* value below 0.05 was considered significant.

## Results

### EphA7 is significantly hypermethylated in CC tissues

**Fig. 1:**
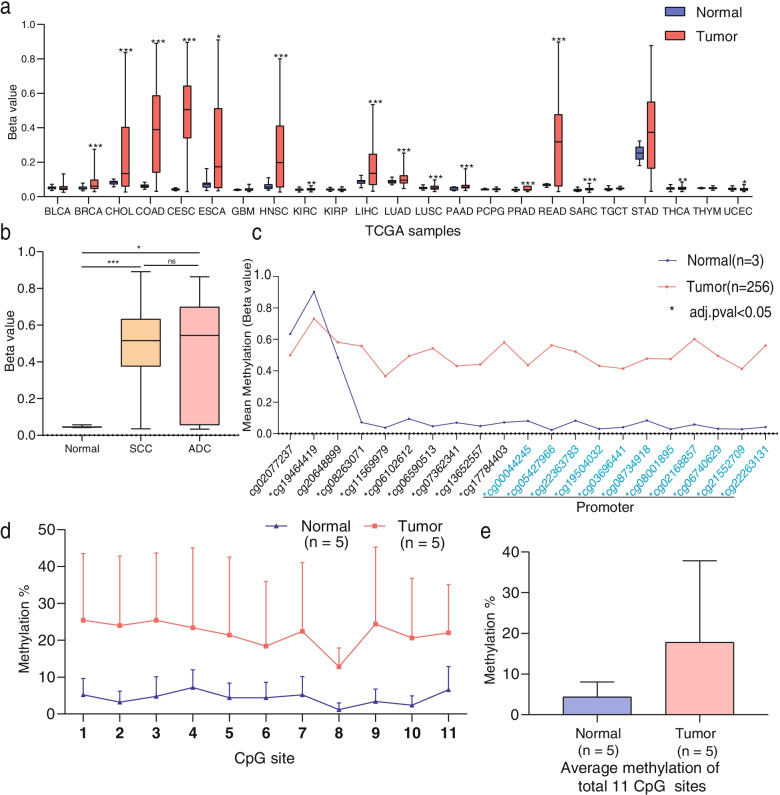
The promoter of EphA7 is significantly hypermethylated in CC tissues. **a** EphA7 hypermethylation was observed in 15 out of 23 categories of cancer tissues compared with adjacent normal tissues (*P* < 0.05) based on the UALCAN web tool, and the median beta value of CESC was the most prominent (△beta value = 0.464, *P* < 0.05). BLCA: Bladder urothelial carcinoma; BRCA: Breast invasive carcinoma; CHOL: Cholangiocarcinoma; COAD: Colon adenocarcinoma; CESC: Cervical squamous cell carcinoma and endocervical adenocarcinoma; ESCA: Esophageal carcinoma; GBM: Glioblastoma multiforme; HNSC: Head and neck squamous cell carcinoma; KIRC: Kidney renal clear cell carcinoma; KIRP: Kidney renal papillary cell carcinoma; LIHC: Liver hepatocellular carcinoma; LUAD: Lung adenocarcinoma; LUSC: Lung squamous cell carcinoma; PAAD: Pancreatic adenocarcinoma; PCPG: Pheochromocytoma and paraganglioma; PRAD: Prostate adenocarcinoma; READ: Rectum adenocarcinoma; SARC: Sarcoma; TGCT: Testicular germ cell tumors; STAD: Stomach adenocarcinoma; THCA: Thyroid carcinoma; THYM: Thymoma; UCEC: Uterine corpus endometrial carcinoma. **b** There was no significant difference in EphA7 methylation between SCC and ADC. **c** The levels of mean EphA7 methylation at a total of 21 probes sites. Among them, all the 11 probes in the promoter region (blue) exhibited significant differences between tumor and adjacent normal specimens (*P* < 0.05). * represents adjusted *P* value < 0.05. Plot and *P* value were produced via Wanderer. **d** Pyrosequencing showed that EphA7 promoter methylation was higher in the tumor tissues compared with normal. **e** Compared with the normal cervical tissues (*n* = 5), the average promoter methylation level of EphA7 within a total of 11 CpG sites was 4.09-fold higher in tumors (*n* = 5) according to pyrosequencing

According to the UALCAN website, the EphA7 promoter was hypermethylated in 15 of 23 categories of cancer (*P* < 0.05) compared with adjacent normal tissues (Fig. 1a). Furthermore, among cancers, the difference of EphA7 hypermethylation in cervical squamous cell carcinoma & endocervical adenocarcinoma (CESC) and normal tissues was the most prominent (△beta value = 0.464, *P* < 0.05); the median beta value was listed in Table. s2. However, there was no significant difference in EphA7 methylation between cervical squamous cell carcinoma (SCC) and cervical adenocarcinoma (ADC) (Fig. 1b).

Therefore, we further analyzed the methylation status of the EphA7 gene from the TCGA database using Wanderer, an online tool. As shown in Fig. 1c, there were a total of 21 HumanMethylation 450 probes located in the EphA7 gene and 11 probes in the promoter region that exhibited significant differences (*P* < 0.05) between tumor tissues and adjacent normal specimens. The differences in in global EphA7 methylation are summarized in Table. s3.

Subsequently, pyrosequencing was performed to quantify the methylation level of EphA7 at each CpG site along the sequence positions of the promoter. Compared with normal cervical tissues, the average methylation level of EphA7 the average methylation level of EphA7 within a total of 11 CpG sites was 4.09-fold higher in tumors (Fig. 1d, e).

### Promoter methylation of EphA7 is inversely correlated with gene expression in CC

**Fig. 2:**
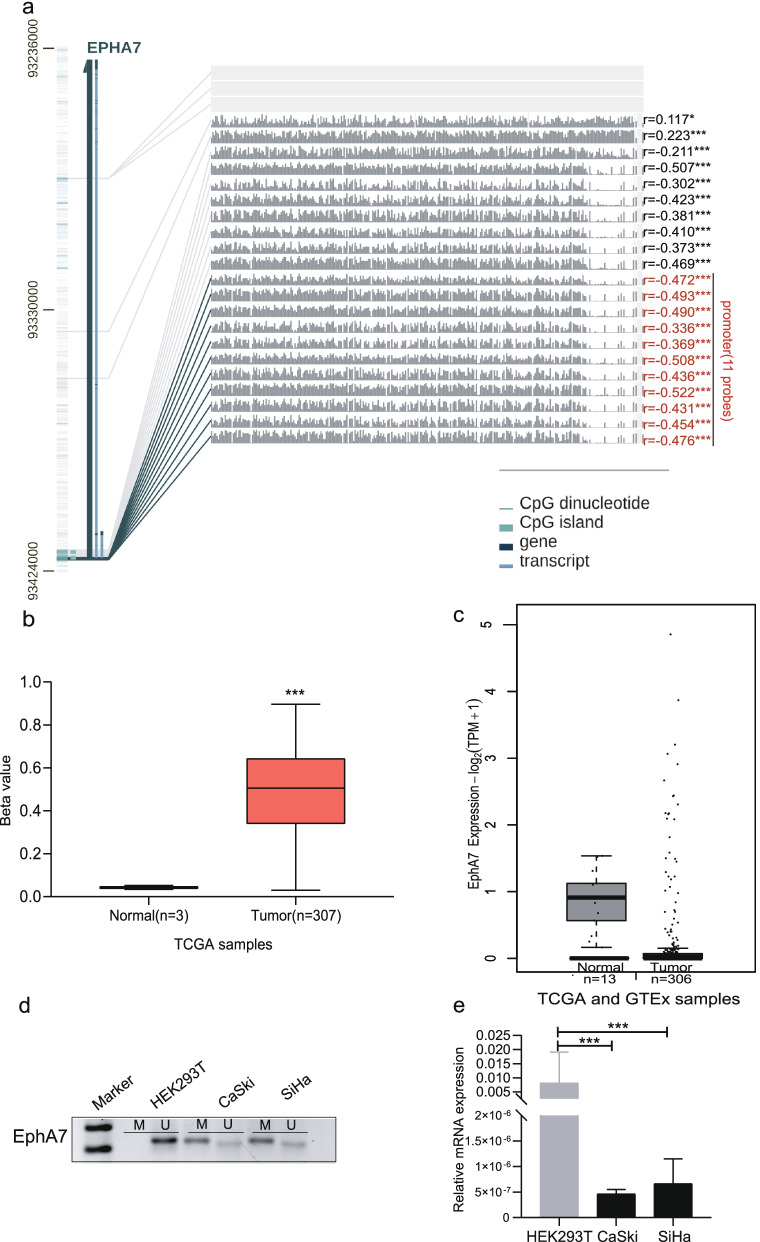
Promoter methylation of EphA7 is inversely correlated with gene expression in CC. **a** Among the 21 HumanMethylation 450 probes, 19 probes showed significantly negative expression (Pearson correlation coefficients from -0.211 to -0.522). Statistical significance was indicated in the right side (* *P* < 0.05,***P < 0.001) and and the promoter probes of EphA7 are highlighted in red color. **b** The EphA7 promoter methylation level was higher in the cancer tissues (*n* = 3) than in adjacent normal (*n* = 307) tissues according to UALCAN. **c** The expression of EphA7 was downregulated in cevical cancer tissues according to GEPIA based on TCGA and GTEx. **d** EphA7 was methylated in CaSki and SiHa cells, and unmethylated in HEK293T cells via MSP. M: methylation, U: unmethylation. Full-length gel pictures are presented in Figure. s2. **e** qRT–PCR showed that the expression level of EphA7 was significantly lower in CaSki and SiHa cells compared with HEK293T cells (*P* < 0.001)

By MEXPRESS web tool, it was observed that 19 out of 21 HumanMethylation 450 probes located in the EphA7 gene showed significantly negative expression (Pearson correlation coefficients from -0.211 to -0.522), and the correlation was more significantly pronounced in the highlighted region, which focused on the EphA7 promoter (Fig. 2a).

This inverse correlation was further confirmed by the UCLCAN and GEPIA web tools. As presented in Fig. 2b and 2c, EphA7 was notably hypermethylated and downregulated in cervical cancer compared with normal tissues (*P* < 0.05).

Consistent with the bioinformatic findings, EphA7 methylation bands that were observed in CaSki and SiHa cells, but not present in HEK293T cells via MSP (Fig. 2d). Full-length gel pictures are presented in Supplementary Figure. s2. However, the expression level of EphA7 was significantly decreased in the CaSki and SiHa cells compared with the HEK293T cells (*P* < 0.001) (Fig. 2e).

### DNA methylation plays a direct role in EphA7 gene regulation

To confirm that DNA methylation affects EphA7 expression directly, a set of CRISPR-based DNA methylation regulation tools (dCas9-Tet1/DNMT3a) was developed to target the EphA7 promoter region.

**Fig. 3:**
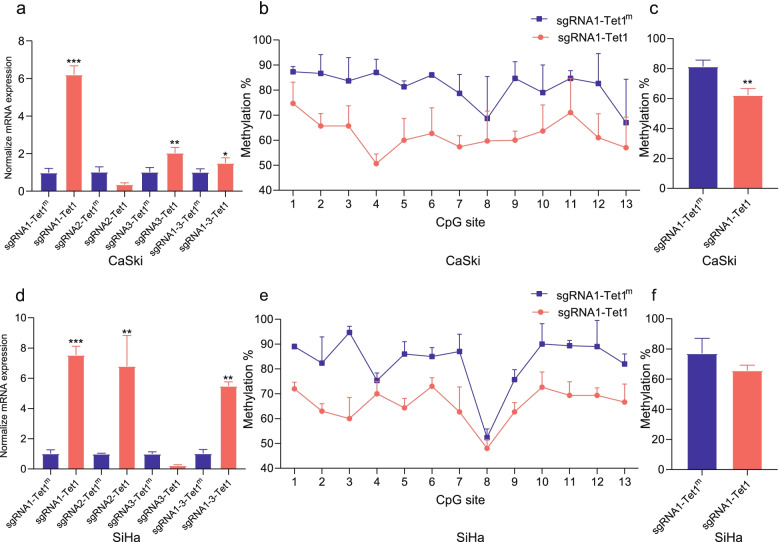
Targeted DNA demethylation of the EphA7 promoter using the dCas9-Tet1 system in CC cell lines. The expression of EphA7 was upregulated at various levels in CaSki (**a**) and SiHa (**d**) cells transfected with combinations of dCas9-Tet1 with sgRNAs compared with the control(Tet1^m^, Tet1^m^ was Tet1 mutations, *n* = 3). Pyrosequencing showed that the EphA7 promoter methylation d was lower in the dCas9-Tet1 group compared with Tet1^m^ in CaSki (**b**) and SiHa (**e**) (*n* = 3).The average methylation level in the promoter region of EphA7 in the sgRNA1 group respectively decrease by 19.10% and 17.23% in CaSki (**c**) and SiHa (**f**) cells, respectively, compared with the control (*n* = 3)

Since EphA7 was hypermethylated in CC cells (CaSki and SiHa), we applied 3 sgRNAs targeted for activation and the dCas9-Tet1 plasmid to upregulate EphA7 expression via demethylation. In CaSki cells, the sgRNA1 (6.20-fold) and sgRNA 3 (2.04-fold) groups had significantly increased EphA7 gene expression compared with the control group (Tet1^m^) (Fig. 3a). However, EphA7 gene expression did not increase further in the group with all three sgRNAs (sgRNA1 + 2 + 3) (1.49-fold). Then, we performed pyrosequencing to detect the methylation level in the promoter region of EphA7 in the sgRNA1 group in CaSki cells. As expected, compared with the control (Tet1^m^) group, the global methylation level decreased, and the average methylation level decreased by 19.10% in CaSki cells (Fig. 3b, c, Table. s4).

In SiHa cells, sgRNA1 (7.54-fold), sgRNA2 (6.80-fold) and sgRNA (all) (5.49-fold) effectively increased EphA7 gene expression (Fig. 3d). In the sgRNA1 group, similar to the results from CaSki, the average methylation level decreased by 17.23% (Fig. 3e, f, Table. s4).

**Fig. 4:**
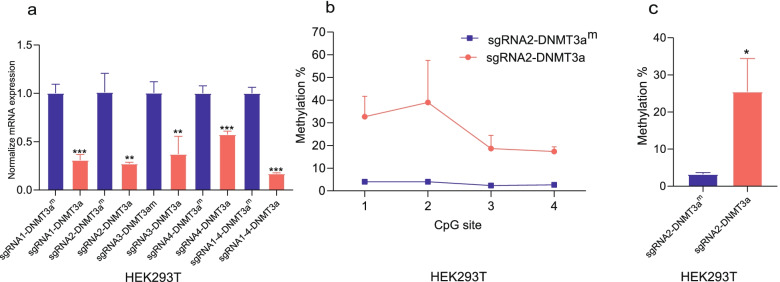
Increased DNA methylation of EphA7 by the dCas9-DNMT3a system. **a** qRT–PCR demonstrated that EphA7 mRNA levels were downregulated compared with DNMT3a^m^ (DNMT3a^m^ was DNMT3a mutations) after cotransfection of 4 repression sgRNAs with the dCas9-DNMT3a in HEK293T cells (*n* = 3). **b** Pyrosequencing showed that the methylation level in the dCas9-DNMT3a group were higher than the control (*n* = 3). **c** The average CpG sites of the promoter increased nearly 23.67% (*n* = 3) in the dCas9-DNMT3a group

Conversely, we selected 4 repression sgRNAs with the dCas9-DNMT3a plasmid to increase methylation and downregulate gene expression in the EphA7 unmethylated HEK293T cells. As expected, the EphA7 mRNA level decreased by 69.05% (sgRNA1 group), 72.75% (sgRNA2 group), 86.23% (sgRNA3 group), 42.50% (sgRNA4 group), and 83.04% (sgRNA1 + 2 + 3 + 4 group) (Fig. 4a). In the sgRNA2 group, the pyrosequencing results showed that the average level CpG sites of the promoter increased by nearly 23.67%. (Fig. 4b, c, Table. s5).

### Validation of the potential clinical value of EphA7 methylation in the diagnosis of CC

**Fig. 5:**
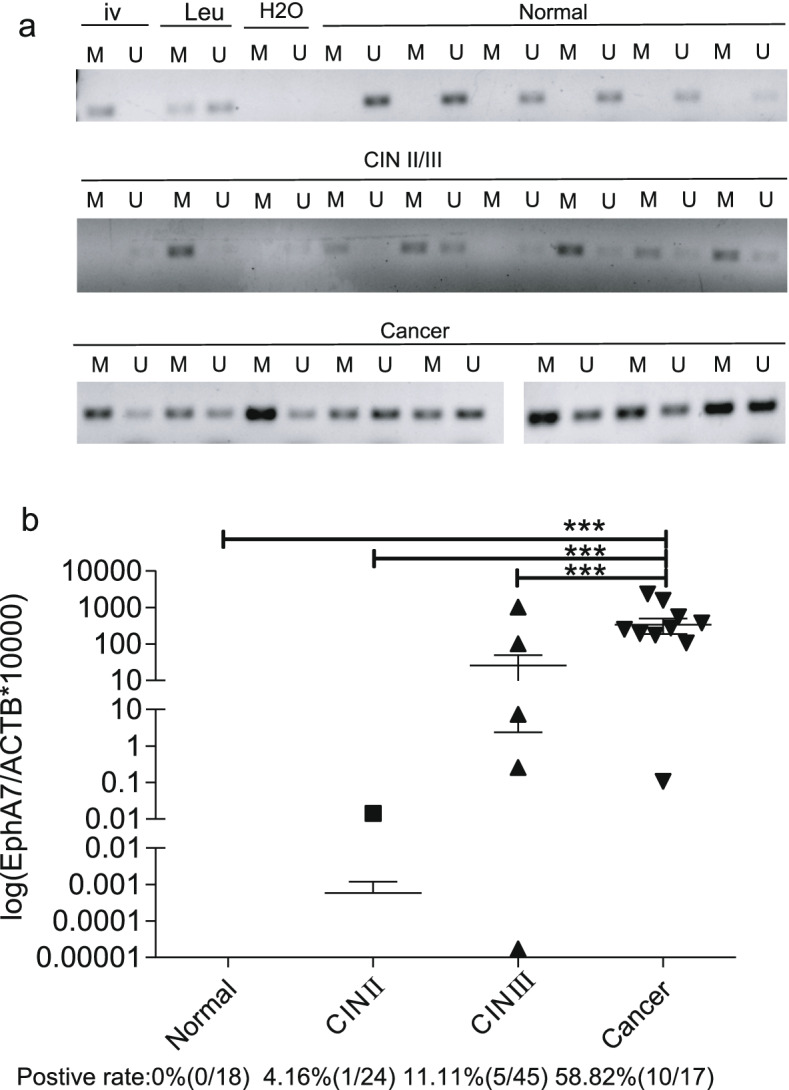
Validation of the potential value of EphA7 methylation for the diagnosis of CC. **a** The methylation-positive rate was 0% (0/25) in normal tissues, 45.83% (11/24) in CIN II/III, and 100% (8/8) in cancer tissues via MSP. Leu: Leukocyte DNA from healthy women was used as a negative control of methylation. iv: In vitro methylated leukocyte DNA was used as a positive control. *Methylation positive results*: the methylated bands were heavier than the unmethylated bands. *Methylation negative results: *the methylated bands were lighter than the unmethylated bands. Full-length gel pictures are presented in Figure. s3. **b** QMSP showed that the methylation level of EphA7 increased with the severity of cervical lesions (H = 27.311, *P* < 0.001)

MSP was performed in 57 cervical tissues, and the methylation positive rate in tissues was 0% (0/25) in normal tissues, 45.83% (11/24) in CINII/III, and 100% (8/8) in cancer tissues (Fig. 5a). Full-length gel pictures are presented in Supplementary Figure. s3.

QMSP were performed on a total of 114 TCT specimens. The results showed that the methylation level increased with the progression of cervical disease (*P* < 0.05), the positive detection rates of methylation were 0% (0/28) in normal tissues, 4.16% (1/24) in CINII, and 11.11% (5/45) in CINIII, and 58.82% (10/17) in cancer (Fig. 5b), and the methylation level of EphA7 increased with the severity of cervical lesions (H = 27.311, *P* < 0.001).

Furthermore, Kaplan–Meier curves demonstrated that lower methylation of EphA7 was correlated with an increased patient survival period via LinkedOmics (Figure. s4a). The Human Protein Atlas also confirmed that high expression of EphA7 was associated with improved survival (Figure. s4b).

## Discussion

Aberrant methylation of EphA7 has been reported in the process of carcinogenesis but not in CC. Our study is the first to systematically investigate the hypermethylation of EphA7 in CC via an effective protocol that combines dry-lab data and wet-lab experiments. Furthermore, CRISPR-dCas9 tools were employed to precisely modify the CpG island in the promoter, which successfully provided direct evidence that EphA7 methylation is involved in its expression.

Rapid developments in bioinformation have inspired suggestions that the field of precision oncology will greatly benefit from a multiomics analytical approach [32–34]. Genomic datasets and web analysis tools have indeed opened the door to deeper and wider exploration of novel biomarkers. However, this novel opportunity also requires creative and skillful solutions to address noisy, unstructured information to offer valuable biological insights. Hence, we developed a logistically pipeline comprising both dry-lab data and wet-lab experiments to uncover EphA7 methylation in CC. First, we investigated EphA7 methylation by applying multiweb tools to data from TCGA and GTEx. TCGA is a public database that includes 33 cancer types and matched clinical data. GTEx is a normal tissue expression dataset that contains RNA-seq profiles of multiple tissues and cell types from hundreds of demographically diverse healthy individuals. However, because the raw data are difficult to interpret, we explored EphA7 hypermethylation in CC tissues using UALCAN and Wanderer web tools. Using MEXPRESS, the methylation level in the promotor region was negatively correlated with the expression, which suggests that EphA7 is a suppressor gene in cervical carcinogenesis. LinkedOmics analysis demonstrated that lower methylation notably increased the patient survival period. After verifying the bioinformatic results in cell lines, we further moved to real tissues and clinical TCT samples, and both confirmed EphA7 hypermethylation in CC.

Additionally, although decades of research have focused on the biological function of DNA methylation, the role of DNA methylation in targeted gene expression remains unclear due to a lack of molecular tools to precisely modify DNA methylation in the genome [35]. The advent of the CRISPR system has enabled precise and stable editing of the epigenome at targeted sgRNAs by fusing catalytically dead Cas9 (dCas9) to DNA methylation-modifying enzymes such as DNA methyltransferases (DNMTs) and Ten-eleven translocation methylcytosine dioxygenases (Tets) [36]. DNMTs target cytosines and facilitate methylation of CpG islands, which contributes to the regulation of gene expression [37]. DNA methylation is maintained by the methyltransferase DNMT1, while DNMT3a and DNMT3b provide de novo CpG methylation [38]. By contrast, Tet promotes the process of DNA demethylation. Both DNMTs and Tets activities play important roles in the dynamic changes in DNA methylation [39, 40]. X.S. Liu et al. [30] constructed the dCas9-Tet1/DNMT3a vector by fusing Tet1 or DNMT3a with the dCas9 protein for re-editing DNA methylation in mice. Moreover, they determined that the CRISPR vector system has better methylation editing efficiency than the TALE vector [30] and that the off-target effect of dCas9-Tet1 on the editing DNA methylation is minimal [41]. Therefore, to establish that DNA methylation plays a direct role in EphA7 expression, both CRISPR-dCas9-Tet1, which targets demethylation, and CRISPR-dCas9-DNMT3a, which targets methylation, were applied in our study. As expected, with the dCas9-Tet1 and sgRNA tools, EphA7 was activated with loss of methylation in the promoters of CaSki (19.10%) and SiHa (17.23%), respectively. Simultaneously, we induced a 23.67% increase in methylation in the first exon of EphA7 via dCas9-DNMT3a, and the expression was indeed downregulated. These results offer strong evidence that EphA7 DNA methylation participates in CC through perturbation of gene regulation.

Furthermore, the efficiency of the CRISPR tools in our study is comparable with the results of other studies. Nozomi Hanzawa et al. [42] reduced DNA methylation by 31.5% and 27.6% with different single sgRNAs. X.S. Liu and J.Wu et al. induced gene hypermethylation of approximately 20–30% using dCas9-DNMT3a [30, 43]. However, Nozomi Hanzawa et al. [42] showed that cotransfected sgRNAs result in additively DNA demethylation levels (approximately 40%) [42], and the efficiency is equal to that of individual sgRNAs when multiple sgRNAs bind in the same region but on the opposite strand, thus serving as competing sgRNAs [44]. Consistent with these results, EphA7 expression was not enhanced more when all the sgRNAs were combined in our study.

Additionally, DNA methylation may disrupt the binding of transcription factors to regulate the regions of target genes [45], or conversely, the binding of transcription factors to these regions may prevent their methylation [46]. In our study, one of the CpG sites of EphA7 presented much lower methylation (Fig. 1d, 3e), with almost no difference between the demethylation group and control. Hence, we investigated the possible disrupted transcription factor via J﻿A﻿SPA﻿R [26]. The results show that Yin Yang 1 (YY1) and transcription factor AP-2 alpha (TFAP2A) are closely related to DNA methylation (Figure. s5). The YY1/polycomb group (PcG) protein/DNMT complex maintains gene methylation and contributes to gene inactivation [47]. DNA methylation can increase the binding of the transcription factor TFAP2A to the target site, leading to suppressed gene expression [48]. These could be the reasons for the low methylation level at the 8th CpG site (Fig. 1d, 3e),but the practical implications require evaluation in a separate study.

Last but not least, SCC and ADC are two major subtypes of CC. Compared with SCC, ADC is mainly diagnosed in more advanced stages and has a worse prognosis. In the last decade, the incidence of ADC has increased rapidly [31]. One reason for the upward trend of ADC is the relatively low effectiveness of cytomorphological detection in screening programs; therefore, novel biomarkers for CC are required that ideally can identify both subtypes. Fortunately, EphA7 is hypermethylated in both SCC and ADC in TCGA datasets and in SiHa (SCC) and CaSki (ADC) cells.

## Conclusions

EphA7 hypermethylation is a potential biomarker for the diagnosis and screening of CC. CRISPR provides a set of powerful tools to investigate the functional significance of DNA methylation in a locus-specific manner. Subsequently, based on the achievements from this study, it’s reasonable to move to the next two directions, one is to further the fundamental research on the detailed mechanism of EphA7 methylation with other cofactors, the other one is to perform a large cohort evaluation that includes all stages from normal to cervical neoplasia, including CINI–CINIII, and cancer to advance the translational research, which both are currently being studied in our laboratory and will be presented in the coming future.

## Supplementary Information

**Additional file 1: Supplemental Table1. **Primer and probe sequence used in this study. **Supplemental Table2. **The median of beta value of EphA7 methylation in normal vs. 23 types of tumor. **Supplemental Table 3. **Each CpG sites of EphA7 methylation in normal vs. CSEC. **Supplemental Table 4.**The average methylation (%) of each CpG site of EphA7 in actsgRNA1 group (dCas9-Tet1 vs. control). **Supplemental Table 5. **The average methylation (%) of each CpG site of EphA7 in repsgRNA2 group in HEK293T cells (dCas9-DNMT3a vs. control). **Figure.s1 **The site of sgRNAs with CRISPR-dCas9 systems targeted in EphA7 promoter and exon 1th.Three active sgRNAs (act-sgRNAs) for demethylation, and four repressive sgRNAs (rep-sgRNAs) for increasing methylation were designed. **Figure.s2 **Full length gel pictures for the results of EphA7 methylation in cell lines using MSP. The marked part was presented in Figure. 2d. **Figure.s3 **Full length gel pictures for the verification results of EphA7 methylation in total 57 cervical tissues using MSP, consisted of 25 normal, 24 CINII/III grade and 8 cancer samples. The marked part was presented in Figure. 5. **Figure.s4 **Kaplan-Meier curve of the survival of patients with high and low levels of EphA7 methylation or expression. **a **Kaplan-Meier curves demonstrated that lower methylation was correlated with an increased patient survival period via LinkedOmics. **b **The high expression of EphA7 was associated with improved survival confirming by Human Protein Atlas. **Figure.s5 **The transcription factors was preditected located in the promoter of EphA7 CpG site. Red “cg” was the target CpG site, which has the possiblity to bind with YY1, TFAP2A via JASPAR.

## Data Availability

All data generated or analysed during this study are included in this published article and its supplementary information files.

## Abbreviations

ADC: Cervical adenocarcinoma
CC: Cervical cancer
CESC: Cervical squamous cell carcinoma & endocervical adenocarcinoma
CIN: Cervical intraepithelial neoplasia
CRISPR: Clustered regularly interspaced short palindromic repeats
DNMT: DNA methyltransferase
GTEx: Genotype Tissue Expression
HEK293T: Human embryonic kidney 293 T
hr-HPV: High-risk human papillomavirus
MSP: Methylation-specific PCR
QMSP: Quantitative methylation-specific PCR
qRT–PCR: Quantitative real-time PCR
SCC: Cervical squamous cell carcinoma
sgRNA: Single guide RNA
TCGA: The Cancer Genome Atlas
TCT: Thinprep cytologic test
Tet: Ten-eleven translocation methylcytosine dioxygenase
TFAP2A: Transcription factor AP-2 alpha
WHO: World Health Organization
5-aza-dc: 5-aza-2-deoxycytidine
YY1: Yin Yang 1
