# Supplementary material for: The identification and validation of EphA7 hypermethylation, a novel biomarker, in cervical cancer
Source: BMC Cancer. 2022 Jun 9;22:636. doi: 10.1186/s12885-022-09653-7 (PMC9185921; doi:10.1186/s12885-022-09653-7)
Supplement: Supplementary file 1 — Additional file 1: Supplemental Table1. Primer and probe sequence used in this study. Supplemental Table2. The median of beta value of EphA7 methylation in normal vs. 23 types of tumor. Supplemental Table 3. Each CpG sites of EphA7 methylation in normal vs. CSEC. Supplemental Table 4.The average methylation (%) of each CpG site of EphA7 in actsgRNA1 group (dCas9-Tet1 vs. control). Supplemental Table 5. The average methylation (%) of each CpG site of EphA7 in repsgRNA2 group in HEK293T cells (dCas9-DNMT3a vs. control). Figure.s1 The site of sgRNAs with CRISPR-dCas9 systems targeted in EphA7 promoter and exon 1th.Three active sgRNAs (act-sgRNAs) for demethylation, and four repressive sgRNAs (rep-sgRNAs) for increasing methylation were designed. Figure.s2 Full length gel pictures for the results of EphA7 methylation in cell lines using MSP. The marked part was presented in Figure. 2d. Figure.s3 Full length gel pictures for the verification results of EphA7 methylation in total 57 cervical tissues using MSP, consisted of 25 normal, 24 CINII/III grade and 8 cancer samples. The marked part was presented in Figure. 5. Figure.s4 Kaplan-Meier curve of the survival of patients with high and low levels of EphA7 methylation or expression. a Kaplan-Meier curves demonstrated that lower methylation was correlated with an increased patient survival period via LinkedOmics. b The high expression of EphA7 was associated with improved survival confirming by Human Protein Atlas. Figure.s5 The transcription factors was preditected located in the promoter of EphA7 CpG site. Red “cg” was the target CpG site, which has the possiblity to bind with YY1, TFAP2A via JASPAR. [file 12885_2022_9653_MOESM1_ESM.pdf]

Supplemental Table1.Primer and probe sequence used in this study

	Primer or Probe	Sequence(5' to 3')	Product size(bp)
**qRT-PCR**	EphA7-F	CAGATTCGGGCTTTTACTGC	187
	EphA7-R	AGCCAAAGACCATGAACACC	
	GAPDH-F	GAAATCCCATCACCATCTTCCAGG	120
	GAPDH-R	GAGCCCCAGCCTTCTCCATG	
**MSP**	EphA7-MF	ATTTGATTTCGTTCGGTATC	229
	EphA7-MR	CTCCGACTACAAACCGACCG	
	EphA7-UF	ATTT GATTTTGTTTGGTATT	229
	EphA7-UR	CTCCAACTACAAACCAACCA	
**QMSP**	EphA7-QMSP-F	ATTTGATTTCGTTCGGTATC	117
	EphA7-QMSP-R	ACCGACCTACCAAAAAACGAT	
	ACTB-QMSP-F	TGGTGATGGAGGAGGTTTAGTAAGT	100
	ACTB-QMSP-R	AACCAATAAAACCTACTCCTCCCTTAA	
	Probe-EphA7	6-FAM-CGAAATCCGAAACAAAAACC-BHQ1	
	Probe-ACTB	6-FAM-ACCACCACCCAACACACAATAACAA ACACA-BHQ1	
**Pyrosequencing**	Tissue-pyro-F	ATTTGGAGGGAATTTTGGATTAG	231
	Tissue-pyro-R	ACTCCACACTCCAATAATATCAATTAA	
	Tissue-pyro-S	ACTCCACACTCCAATAATATCAATTAA	
	Act-pyro-F	ATTTGGAGGGAATTTTGGATTAG	234
	Act-pyro-R	CTTACTCCACACTCCAATAATATCA	
	Act-pyro-S	AGGGAATTTTGGATTAGTAA	
	Rep-pyro-F	AGAAGGGGAATAGATGTAAGGAGTATAA	238
	Rep-pyro-R	AAACTTTACTTTCCCATCACCTTACC	

Supplemental Table2. The median of beta value of EphA7 methylation in normal vs. 23 types of tumor

Type	Normal beta value	CESC beta value	Difference value	*P* value
CESC	0.042	0.506	0.464	<0.001
COAD	0.060	0.390	0.330	<0.001
READ	0.072	0.318	0.246	<0.001
HNSC	0.057	0.199	0.142	<0.001
STAD	0.253	0.374	0.121	0.447
ESCA	0.073	0.174	0.101	<0.05
CHOL	0.084	0.134	0.050	<0.001
LIHC	0.090	0.136	0.046	<0.001
BRCA	0.047	0.061	0.014	<0.001
LUAD	0.086	0.096	0.010	<0.001
SARC	0.036	0.043	0.007	<0.001
PAAD	0.052	0.057	0.005	<0.001
TGCT	0.043	0.047	0.004	0.814
KIRC	0.041	0.043	0.002	<0.01
LUSC	0.049	0.051	0.002	<0.001
PCPG	0.040	0.042	0.002	0.253
THCA	0.046	0.048	0.002	<0.01
PRAD	0.040	0.041	0.001	<0.001
GBM	0.040	0.040	0.000	0.627
KIRP	0.042	0.041	-0.001	0.134
THYM	0.050	0.049	-0.001	0.862
UCEC	0.045	0.042	-0.003	<0.05
BLCA	0.054	0.049	-0.005	0.061

CESC:Cervical squamous cell carcinoma and endocervical adenocarcinoma;BLCA: Bladder urothelial carcinoma; BRCA:Breast invasive carcinoma;CHOL: Cholangiocarcinoma; COAD:Colon adenocarcinoma; ESCA:Esophageal carcinoma; GBM:Glioblastoma multiforme; HNSC:Head and Neck squamous cell carcinoma; KIRC:Kidney renal clear cell carcinoma;KIRP:Kidney renal papillary cell carcinoma; LIHC:Liver hepatocellular carcinoma;LUAD:Lung adenocarcinoma; LUSC:Lung squamous cell carcinoma; PAAD:Pancreatic adenocarcinoma; PCPG:Pheochromocytoma and Paraganglioma;PRAD:Prostate adenocarcinoma; READ:Rectum adenocarcinoma;SARC:Sarcoma; TGCT:Testicular Germ Cell Tumors; STAD:Stomach adenocarcinoma; THCA:Thyroid carcinoma;THYM:Thymoma; UCEC:Uterine Corpus Endometrial Carcinoma.

Supplemental Table 3.Each CpG sites of EphA7 methylation in normal vs. CSEC

Probe	Normal beta value	CESC beta value	Difference value	adj.pval
**cg02168857**	0.058	0.601	0.542	0.015
**cg05427966**	0.023	0.562	0.538	0.015
**cg22263131**	0.042	0.556	0.514	0.015
**cg17784403**	0.071	0.583	0.511	0.015
**cg06590513**	0.047	0.541	0.495	0.015
**cg08263071**	0.071	0.559	0.487	0.015
**cg06740629**	0.031	0.493	0.462	0.015
**cg08001895**	0.028	0.473	0.445	0.015
**cg22363783**	0.082	0.523	0.441	0.015
**cg06102612**	0.095	0.492	0.397	0.015
**cg19504032**	0.030	0.427	0.397	0.015
**cg13652557**	0.048	0.444	0.396	0.018
**cg08734918**	0.083	0.478	0.395	0.015
**cg21552709**	0.028	0.409	0.381	0.015
**cg03696441**	0.040	0.41	0.37	0.018
**cg07362341**	0.070	0.433	0.363	0.015
**cg00044245**	0.081	0.436	0.355	0.015
**cg11569979**	0.037	0.368	0.331	0.015
**cg20648899**	0.485	0.585	0.1	0.35
**cg19464419**	0.902	0.733	-0.169	0.031
**cg02077237**	0.634	0.502	-0.132	0.245

The difference value indicated that the beta value of cancer minus the normal. The adj.pval represents adjusted *P*-value.

Supplemental Table 4.The average methylation(%) of each CpG site of EphA7 in act-sgRNA1 group (dCas9-Tet1 vs. control)

Cell lines	CaSki			SiHa		
CpG site	Control	dCas9-Tet1	Demethylation	control	dCas9-Tet1	Demethylation
1	87.33	74.67	12.66	89.00	72.00	17.00
2	86.67	65.67	21.00	82.33	63.00	19.33
3	83.67	65.67	18.00	94.67	60.00	34.67
4	87.00	50.67	36.33	75.33	70.00	5.33
5	81.33	60.00	21.33	86.00	64.33	21.67
6	86.00	62.67	23.33	85.00	73.00	12.00
7	78.67	57.33	21.34	87.00	62.67	24.33
8	68.67	59.67	9.00	52.33	48.00	4.33
9	84.67	60.00	24.67	75.67	62.67	13.00
10	79.00	63.67	15.33	90.00	72.67	17.33
11	84.67	71.00	13.67	89.33	69.33	20.00
12	82.67	61.00	21.67	89.00	69.33	19.67
13	67.00	57.00	10.00	82.00	66.67	15.33
Mean	81.33	62.23	19.10	82.90	65.67	17.23

Supplemental Table 5.The average methylation(%) of each CpG site of EphA7 in rep-sgRNA2 group in HEK-293T cells(dCas9-DNMT3a vs. control)

CpG site	1	2	3	4	Mean
Control	32.67	39.00	18.67	17.33	3.65
dCas9-DNMT3a	32.67	4.00	2.33	2.67	26.92
Increased methlation	28.67	35.00	16.34	14.66	23.67

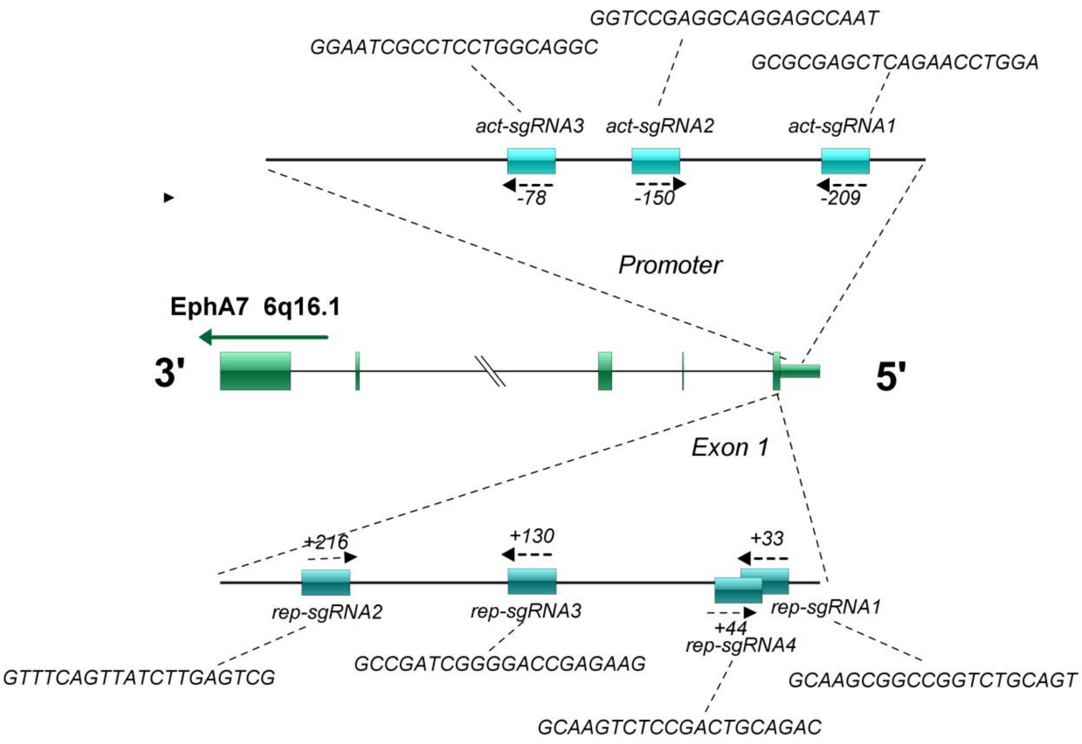

Figure.s1 The site of sgRNAs with CRISPR-dCas9 systems targeted in EphA7 promoter and exon 1th.Three active sgRNAs (act-sgRNAs) for demethylation,and four repressive sgRNAs (rep-sgRNAs) for increasing methylation were designed.

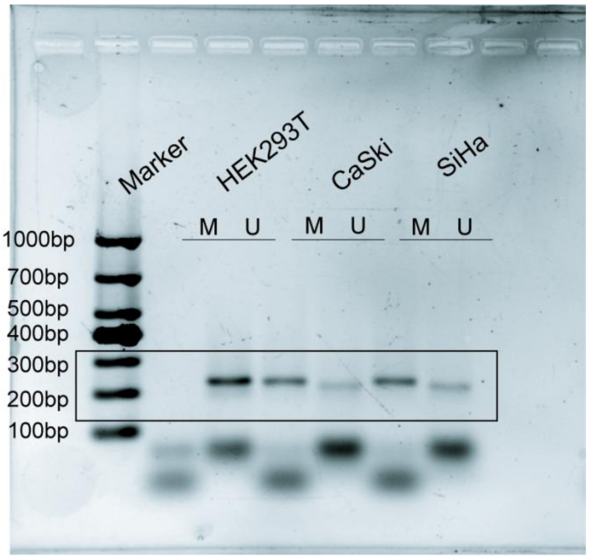

Figure.s2 Full length gel pictures for the results of EphA7 methylation in cell lines using MSP.The marked part was presented in Figure. 2d.

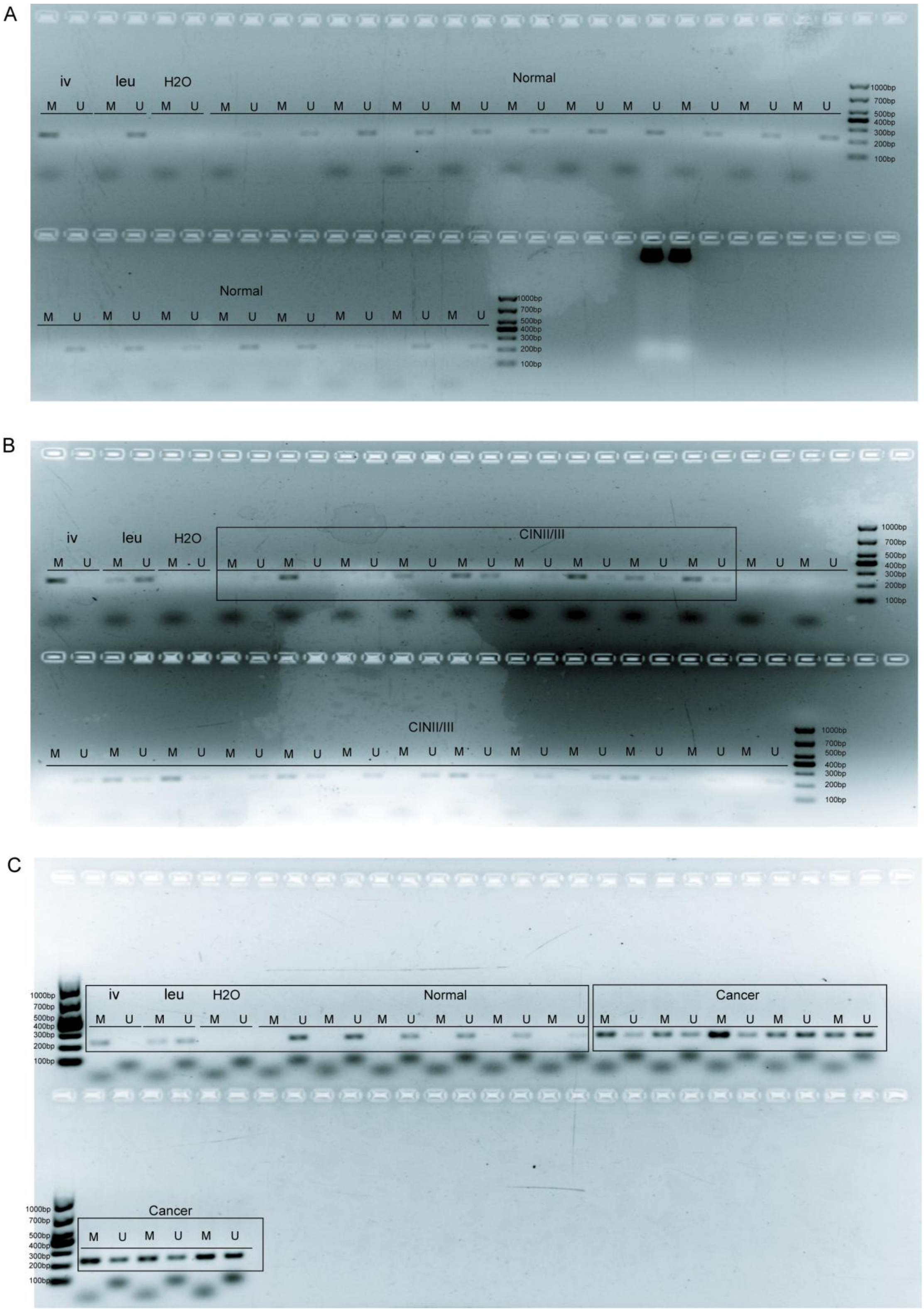

Figure.s3 Full length gel pictures for the verification results of EphA7 methylation in total 57 cervical tissues using MSP, consisted of 25 normal, 24 CINII/III grade and 8 cancer samples.The marked part was presented in Figure. 5.

a

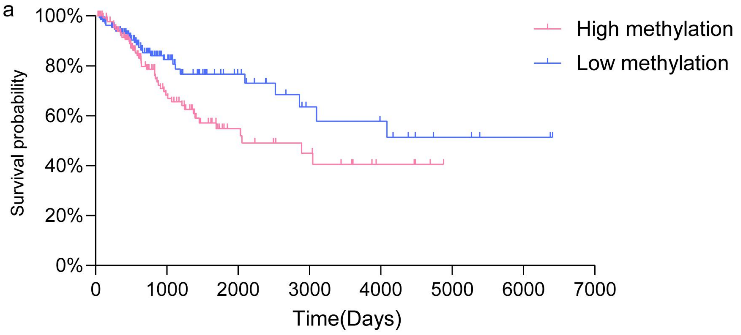

b

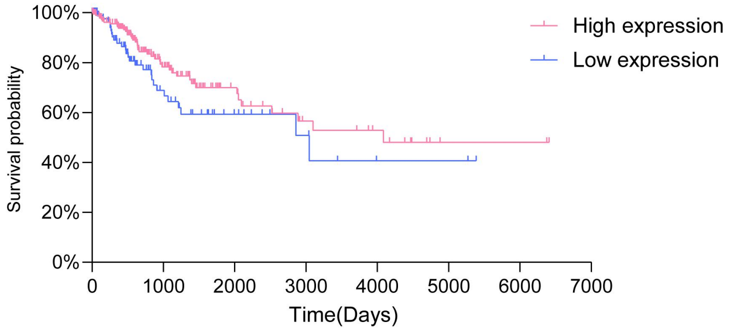

Figure.s4 Kaplan-Meier curve of the survival of patients with high and low levels of EphA7 methylation or expression. (**a**) Kaplan-Meier curves demonstrated that lower methylation was correlated with an increased patient survival period via LinkedOmics. (**b**) The high expression of EphA7 was associated with improved survival confirming by Human Protein Atlas.

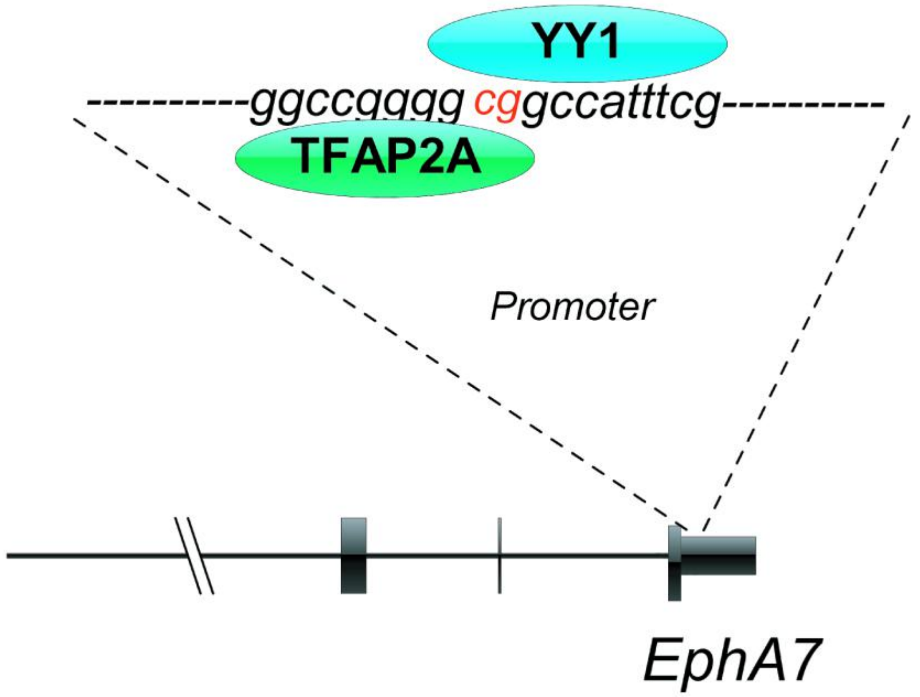

Name	Score	Start	End	Strand	Predicted sequence
YY1	12.752	1870	1881	-	CGAAATGGCCGC
TFAP2A	8.749	1857	1865	-	GCCACGGGG

Figure.s5 The transccription factors was preditected loacted in the promoter of EphA7 CpG site.Red “cg” was the target CpG site, which has the possiblity to bind with YY1,TFAP2A via JASPAR.
